# A splice site mutation in the *FvePHP* gene is associated with leaf development and flowering time in woodland strawberry

**DOI:** 10.1093/hr/uhac249

**Published:** 2022-11-10

**Authors:** Baotian Wang, Weijia Li, Kexin Xu, Yingying Lei, Di Zhao, Xue Li, Junxiang Zhang, Zhihong Zhang

**Affiliations:** Liaoning Key Laboratory of Strawberry Breeding and Cultivation, College of Horticulture, Shenyang Agricultural University, Shenyang, 110866, China; Laboratory of Protected Horticulture (Shenyang Agricultural University), Ministry of Education, Shenyang, People’s Republic of China; Liaoning Key Laboratory of Strawberry Breeding and Cultivation, College of Horticulture, Shenyang Agricultural University, Shenyang, 110866, China; Laboratory of Protected Horticulture (Shenyang Agricultural University), Ministry of Education, Shenyang, People’s Republic of China; Institute of Carbon Materials Science, Shanxi Datong University, Datong, 037009, China; Liaoning Key Laboratory of Strawberry Breeding and Cultivation, College of Horticulture, Shenyang Agricultural University, Shenyang, 110866, China; Laboratory of Protected Horticulture (Shenyang Agricultural University), Ministry of Education, Shenyang, People’s Republic of China; Liaoning Key Laboratory of Strawberry Breeding and Cultivation, College of Horticulture, Shenyang Agricultural University, Shenyang, 110866, China; Laboratory of Protected Horticulture (Shenyang Agricultural University), Ministry of Education, Shenyang, People’s Republic of China; Analytical and Testing Center, Shenyang Agricultural University, Shenyang, 110866, China; Liaoning Key Laboratory of Strawberry Breeding and Cultivation, College of Horticulture, Shenyang Agricultural University, Shenyang, 110866, China; Laboratory of Protected Horticulture (Shenyang Agricultural University), Ministry of Education, Shenyang, People’s Republic of China; Liaoning Key Laboratory of Strawberry Breeding and Cultivation, College of Horticulture, Shenyang Agricultural University, Shenyang, 110866, China; Laboratory of Protected Horticulture (Shenyang Agricultural University), Ministry of Education, Shenyang, People’s Republic of China; Liaoning Key Laboratory of Strawberry Breeding and Cultivation, College of Horticulture, Shenyang Agricultural University, Shenyang, 110866, China; Laboratory of Protected Horticulture (Shenyang Agricultural University), Ministry of Education, Shenyang, People’s Republic of China; Analytical and Testing Center, Shenyang Agricultural University, Shenyang, 110866, China

## Abstract

Leaves and flowers are crucial for the growth and development of higher plants. In this study we identified a mutant with narrow leaflets and early flowering (*nlef*) in an ethyl methanesulfonate-mutagenized population of woodland strawberry (*Fragaria vesca*) and aimed to identify the candidate gene. Genetic analysis revealed that a single recessive gene, *nlef*, controlled the mutant phenotype. We found that FvH4_1g25470, which encodes a putative DNA polymerase α with a polymerase and histidinol phosphatase domain (PHP), might be the candidate gene, using bulked segregant analysis with whole-genome sequencing, molecular markers, and cloning analyses. A splice donor site mutation (C to T) at the 5′ end of the second intron led to an erroneous splice event that reduced the expression level of the full-length transcript of *FvePHP* in mutant plants. FvePHP was localized in the nucleus and was highly expressed in leaves. Silencing of *FvePHP* using the virus-induced gene silencing method resulted in partial developmental defects in strawberry leaves. Overexpression of the *FvePHP* gene can largely restore the mutant phenotype. The expression levels of *FveSEP1*, *FveSEP3*, *FveAP1*, *FveFUL*, and *FveFT* were higher in the mutants than those in ‘Yellow Wonder’ plants, probably contributing to the early flowering phenotype in mutant plants. Our results indicate that mutation in *FvePHP* is associated with multiple developmental pathways. These results aid in understanding the role of DNA polymerase in strawberry development.

## Introduction

Strawberry is a small fruit crop with high economic value and is widely cultivated around the world [1]. Flowering time is a crucial factor in strawberry production, and elucidating the molecular mechanism of strawberry flowering can lay the theoretical foundation for breeding new cultivars. In this study we identified a mutant with narrow leaflets and early flowering (*nlef*) phenotypes in woodland strawberry (*Fragaria vesca*).

Leaves are derived from the shoot apical meristem (SAM), which plays a crucial role in plant growth and development [2, 3]. Leaves store energy and are involved in processes such as photosynthesis, gas exchange, plant architecture determination, thermoregulation, and environmental adaptation in higher plants [4]. Leaf characteristics such as leaf shape, leaf size, and leaf margin affect plant growth. Internal and external factors influence the process of determination of leaf characteristics during leaf development. The internal factors include hormones, genes, and small RNAs [5–7], and the external environmental factors include light, temperature, and humidity [8]. Several recent studies have focused on leaf shape in several crops, such as rice, maize, tomato, and wheat [9–14]. For instance, a study reported that tomato fruit quality and cultivar performance can be predicted based on leaf shape [15]. However, the relationship between leaf shape and agronomic traits still needs to be explored.

Flowers are also derived from the SAM and are crucial for plant reproduction. The yield of many crops is closely related to the flowering time, and the regulation of flowering time involves a complex signaling network influenced by both external factors, such as the photoperiod and vernalization, and internal factors, such as the gibberellin pathway and the autonomous flowering pathway [16, 17]. These pathways influence the expression of flowering-related genes such as *FLOWERING LOCUS T* (*FT*), *APETALA 1* (*AP1*), and *LEAFY* (*LFY*), and regulate flowering [17]. In addition, epigenetic components such as chromatin remodeling and histone modifications are involved in floral transition [18].

**Figure 1:**
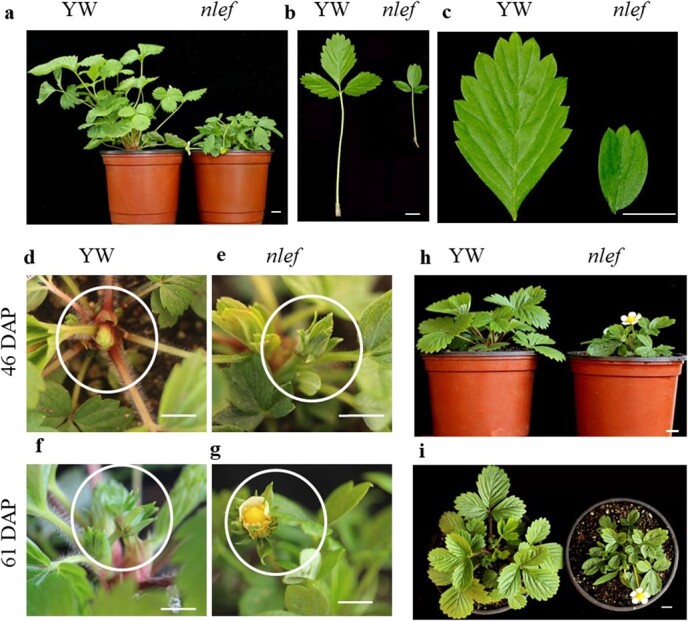
Phenotypic characteristics of YW and *nlef* mutant plants. **a**–**c** Plant phenotypes and leaf characteristics of YW and *nlef*. Plants at 70 DAP. **d**–**g** Flower bud formation in YW and mutant plants observed at 46 DAP (**d**, **e**) and 61 DAP (**f**, **g**). Flower buds are marked with white circles. **h**, **i** Flower bud formed in YW and *nlef*. Scale bar = 1 cm (**a**, **b**, **h**, **i**), 0.5 cm (**c**–**g**).

**Figure 2:**
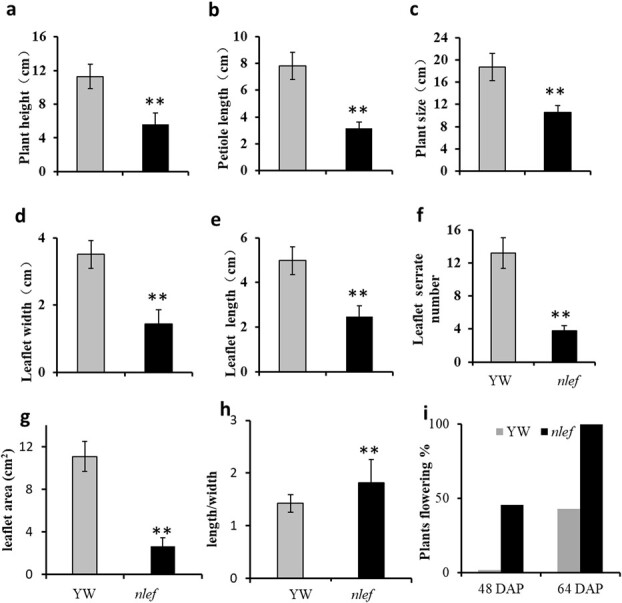
Morphological indices and flowering time of YW and *nlef* mutant. **a**–**h** Plant height, petiole length, plant size, leaflet width, leaflet length, serration number, leaflet area, and leaflet length/leaflet width ratio of YW and *nlef* mutant; *n* = 10 individual plants. **i** Percentage of plants flowering 48 and 64 DAP under mid-day conditions (12 hours light/12 hours dark)*. n* = 70. Statistical significance was determined using Student’s *t*-test in different samples: **P < .01. Error bars represent standard deviations.

In the post-transcriptional process, alternative splicing produces multiple mRNAs from a single pre-mRNA, generating various protein sequences with various activities and new proteins [19]. In higher plants, alternative splicing plays an important role in the response to environmental stimuli and regulates plant development processes such as seed germination and plant flowering [19, 20]. Alternative splicing is classified into five types with differing patterns: intron-retained, exon skipping, alternative 5′/3′ splice sites, alternative first/end exons, and mutually exclusive exons [21]. During the splicing process, the spliceosome splits the intron by identifying the splice sites and the sequence in the 5′ region of the intron to splice the corresponding exon sequence. Mutations in the splice sites exert a major influence on splicing events. For example, an alternative splice site mutation in the third subunit of a DNA polymerase accelerates flowering in *Brachypodium distachyon* by promoting the expression of flowering-related genes [22]. In *Arabidopsis thaliana*, two DNA polymerases, AtPOLH and AtREV1, play a key role in DNA replication and are regulated by alternative splicing [23]. In cucumber, exon skipping in *CsSEP2* affects floral organs and fruit development [24].

In this study, using bulked segregant analysis (BSA) with whole-genome sequencing, we identified an SNP mutation that located the splice site of the *FvePHP* gene and is associated with leaf shape and flowering regulation in woodland strawberry.

## Results

### Phenotypic characterization of the *nlef* mutant

Early-flowering mutants are key resources that facilitate the study of flowering mechanisms. In this study we showed that an *nlef* mutant in an ethyl methanesulfonate-mutagenized *M*_2_ population of woodland strawberry accession ‘Yellow Wonder’ (YW) (Fig. 1). Overall plant size, as measured by diameter and height, are decreased in the *nlef* mutant. The leaflet length, leaflet width, and the length of petioles, number of serrations, and leaflet area are also decreased in the mutant. The ration of leaflet length to leaflet width is increased in the mutant, which indicates that the leaflets of the mutant are narrow (Supplementary Data Table S4, Fig. 2a–g). These data suggest that the overall plant size of the mutant is smaller than that of YW.

To observe and record the flowering of *nlef* plants*,* YW and *nlef* plants were planted under strict chamber conditions. As Fig. 2i shows, the mutant strawberry *nlef* bloomed earlier than YW. On the 64th day after planting (DAP) 100% of mutant strawberry plants had bloomed, whereas at this time 42% of the YW had bloomed. Thus, an early-flowering phenotype was also observed in the mutant, besides the obvious defects in both leaf shape and morphological structure.

### Leaf epidermal cells of ‘Yellow Wonder’ and *nlef* mutant were observed by scanning electron microscopy

**Figure 3:**
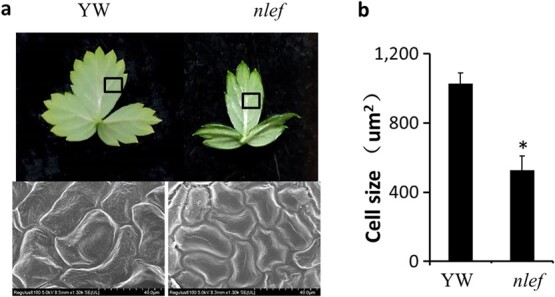
Scanning electron microscopy of leaf epidermal cells of YW and *nlef* mutant. **a** Selected leaf region (top) and cell features (bottom). Black boxes indicate the region used for scanning electron microscopy observation. **b** Cell size of abaxial epidermal cells in YW and *nlef* mutant. Three leaves were selected from YW and *nlef* mutant, and 50 cells were measured for each leaf. Error bars represent standard deviations (*n* = 50). ^*^P < .05 (Student’s *t*-test).

Since cell size and division play crucial roles in plant organ development, we observed epidermal cells of young leaves of YW and the *nlef* mutant using scanning electron microscopy. The results indicated that the abaxial epidermal cells of *nlef* mutant were significantly smaller than those of YW (Fig. 3).

### Genetic analysis of *nlef*

To identify the candidate gene for the *nlef* phenotype, the *nlef* mutant as the female parent was hybridized with the woodland strawberry accession ‘Ruegen’ (RG) to generate an *F*_1_ generation. Then, an *F*_2_ population was obtained by self-pollination of *F*_1_, which included 277 RG plants and 97 *nlef* plants. The ratio of RG plants to *nlef* plants was ~3:1 (χ^2^ = 0.128; χ^2^_0.05_ = 3.84), indicating that the *nlef* phenotype was controlled by a single recessive locus.

**Table 1:** Summary of candidate associated regions observed by the ED method.

Variant	Chromosome_ID	Start (bp)	End (bp)	Size (Mb)	No. of genes
SNP	Fvb1	11 620 000	24 230 000	12.61	1116
InDel	Fvb1	11 570 000	21 380 000	9.81	958
	Fvb1	21 980 000	24 230 000	2.25	133

### Identification of candidate region based on bulk segregant analysis sequencing

Through genome sequencing of RG, *nlef* mutant, and two DNA pools from 37 mutants and 37 RG plants of the *F*_2_ population, we obtained 51 920 640, 19 635 770, 45 181 986, and 52 513 370 high-quality reads, respectively. The Q30 quality scores for the clean reads were 90.88%, 94.29%, 93.35%, and 94.15%, respectively. The sequencing depths of the two parental lines and the two bulks were 57×, 22×, 49×, and 58×, respectively. The mapping rates of clean reads in RG, *nlef*, and the two DNA pools were 95.85%, 97.23%, 95.21%, and 97.21%, respectively.

**Figure 4:**
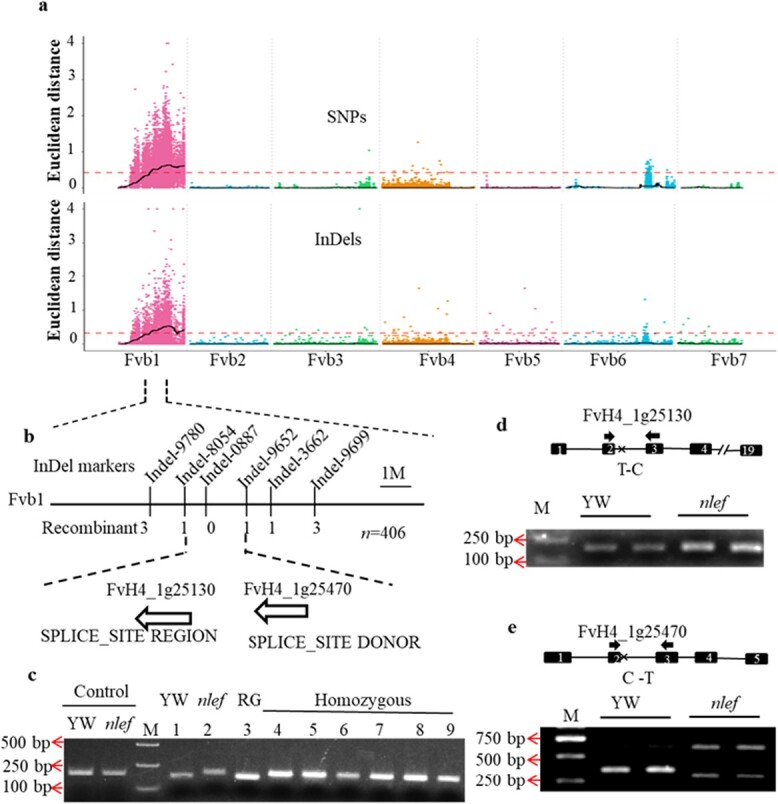
Location and identification of candidate genes. **a** ED value distribution of SNPs and InDels on chromosomes. The *x*-axis shows the chromosome name. Colored points represent the ED value. The black line represents the fitted ED value, and the red dotted line represents the significance correlation threshold. **b** InDel markers used to screen candidate genes. The horizontal line represents chromosome Fvb1 and the vertical line represents the location of the InDel marker. Two splice-site mutant genes are shown at the candidate interval. **c** The dCAPs marker was used to analyze the candidate gene FvH4_1g25470. PCR products were digested with SmaI and detected by 3% agarose gel electrophoresis (lanes 1–9). Control samples were not digested with SmaI. SNP 17218907 was C:C genotype in YW and RG plants, T:T genotype was in *nlef* and six recombinant individuals. ‘Homozygous’ refers to the homozygous SNP mutant (position 17 218 907 C–T) of gene FvH4_1g25470 from the six recombinant individuals with T:T genotype. ‘Heterozygous’ refers to the T:C genotype (position 17 218 907) **d**, **e** Two splice site mutations were detected by 1.5% agarose gel electrophoresis. The position of primer amplification is indicated by the arrow. Exons are represented by black boxes, and the order of exons is shown in the black box. Introns are represented by black lines.

To identify the variation in SNPs and InDels, GATK software (GATK, https://software.broadinstitute.org/gatk/) was used to detect and annotate the SNPs and InDels. In total, we detected 82 695 SNPs and 26 865 InDels in the two parental plants, and 19 099 SNPs and 12 600 InDels in the two bulks. After filtering out low-quality reads, the Euclidean distance (ED) was calculated for all high-quality SNPs and InDels. The thresholds were represented by the median values +3 standard deviations for the ED method. Based on the BSA sequencing results, the candidate regions were observed to be located on chromosome (Chr) Fvb1, which contains 2207 genes (Table 1). When the thresholds were 0.42 and 0.33 for the SNPs and InDels, respectively, Chr Fvb1 was screened from 11.57 to 24.23 Mb. The highest ED value for SNPs and InDels appeared around the regions of 17.79 and 19.30 Mb, respectively (Table 1, Fig. 4a).

We further identified different mutant SNPs or InDels that were homozygous on Chr Fvb1 between ‘*nlef*’ and ‘*nlef-*pool’. The results showed 20 SNPs located on Chr Fvb1 from 12.99 to 24.15 Mb and 19 InDels located on Chr Fvb1 from 13.09 to 19.95 Mb (Supplementary Data Table S2).

To narrow the candidate interval, we increased the number of *F*_2_ individuals from other five *F*_1_ plants. Six InDel markers were developed to test for their linkage to the mutant locus by genotyping 406 recessive individuals from the *F*_2_ individuals from other five *F*_1_ plants (Fig. 4b). Subsequently, the candidate region was delimited to the 1.94 Mb region between 16.30 and 18.24 Mb (Fig. 4b).

**Table 2:** Location, annotation, and nucleotide types of SNPs and InDels in different samples.

Variant type	Gene_ID	Position	Reference genome Hawaii 4	Alternate nucleotides	*nlef*	RG pool	*nlef* pool	RG	Effect	NR_annotation
SNPs	FvH4_1g24520	16 362 657	C	T	0,6	5,3	0,15	9,0	UPSTREAM	ABC transporter G family member 5
	FvH4_1g24550	1 383 420	G	A	0,23	25,15	0,36	34,0	DOWNSTREAM	
	FvH4_1g24610	16 435 257	C	T	0,14	25,8	0,33	33,0	DOWNSTREAM	Uncharacterized protein
	FvH4_1g25060	16 859 493	C	T	0,6	22,9	0,27	39,0	UPSTREAM	Double-stranded RNA-binding protein 4-like
	FvH4_1g25070	16 877 044	C	T	0,17	29,18	0,41	41,0	INTRON	Probable glucan 1,3-α-glucosidase
	FvH4_1g25130	16 923 573	A	G	0,15	24,14	0,43	44,0	SPLICE_SITE_REGION	β-Galactosidase 10
	FvH4_1g25390	17 148 690	A	G	0,13	35,14	0,39	57,0	INTRON	Translation initiation factor eIF-2B subunit γ
	FvH4_1g25470	17 218 907	C	T	0,19	32,12	0,37	53,0	SPLICE_SITE_DONOR	Uncharacterized protein
	N	17 808 192	C	T	0,13	19,7	0,35	63,0	INTRAGENIC	
InDels	FvH4_1g25110	16 904 139	ACTCTCTCTCTCTCTCT	ACTCTCTCTCT	0,10	10,3	0,9	24,1	UPSTREAM	Protein XAP5 circadian timekeeper
	FvH4_1g25400	17 153 065	ATGTGTGTGTGTG	ATGTGTGTGTG	0,10	32,9	0,18	39,2	INTRON	Uncharacterized protein
	FvH4_1g25840	17 748 337	AATATATATATATATATATATAT	AATATATATATATATATAT	0,3	19,5	0,4	45,0	UPSTREAM	ABC transporter D family member 1
	FvH4_1g25850	17 767 424	GAAAA	GAA	0,8	13,2	0,11	25,0	UPSTREAM	Chloride channel protein CLC-c

‘Alternate nucleotides’ refers to mutated bases or sequences. The *nlef* and *nlef* pool represent mutant plants and *F*_2_ population with *nelf* genotype plants, respectively. RG and RG pool refer to RG and *F*_2_ population with RG genotype plants, respectively. The numbers in columns headed *nlef, nlef* pool, RG, and RG pool represent read depths. ‘Effect’ represents genome location information of variant nucleotides or sequences. N was no read support. ‘NR_annotation’ represents genes that were annotated using the NCBI database.

### Identification of the candidate gene

In this study a total of 203 genes were annotated in the 1.94-Mb candidate region, and 3431 SNPs and 887 InDels were contained in this candidate region. According to the annotation of SNPs and InDels, ~33% of SNPs/InDels were located upstream, ~23% were downstream of the genes, ~31% in the intergenic regions, ~16% in the introns, and only ~4.2% of the SNPs and ~ 0.3% of the InDels were located in the coding sequence (CDS) regions (Supplementary Data Table S5). On further analysis, we found that five homozygous mutations were located upstream of the genes shown in Table 2 and two homozygous mutations were located downstream of the genes (Table 2). In addition, three homozygous mutations were located in the introns of genes and one homozygous mutation was located in the intergenic region. None of these mutations changed the CDS. Two SNPs located in splice sites caught our attention (Table 2). The splice site plays a crucial role in the resulting protein sequence. Therefore, we investigated whether the genes themselves give rise to the splicing event. RT–PCR was used to detect splicing events and no distinction was observed in FvH4_1g25130 between YW and the *nlef* mutant, while FvH4_1g25470 in YW and the *nlef* mutant had one and two bands in the gel electrophoresis, respectively (Fig. 4d and e). In addition, RNA-seq of YW and the *nlef* mutant showed that FvH4_1g25470 was the only gene that displayed significantly differential expression between YW and the *nlef* mutant in the 1.94 Mb candidate regions [log_2_FC < −1, false discovery rate (FDR) < 0.05; Supplementary Data Table S3, Supplementary Data Fig. S1].

A dCAPs marker of FvH4_1g25470 was developed based on (pos 17,218,907 C-T,). The result showed that SNP 17,218,907 of FvH4_1g25470 co-segregated with the mutation phenotype in 6 recombinant individuals between the region Indel-9780 and Indel-9699 (Fig. 4c).

**Figure 5:**
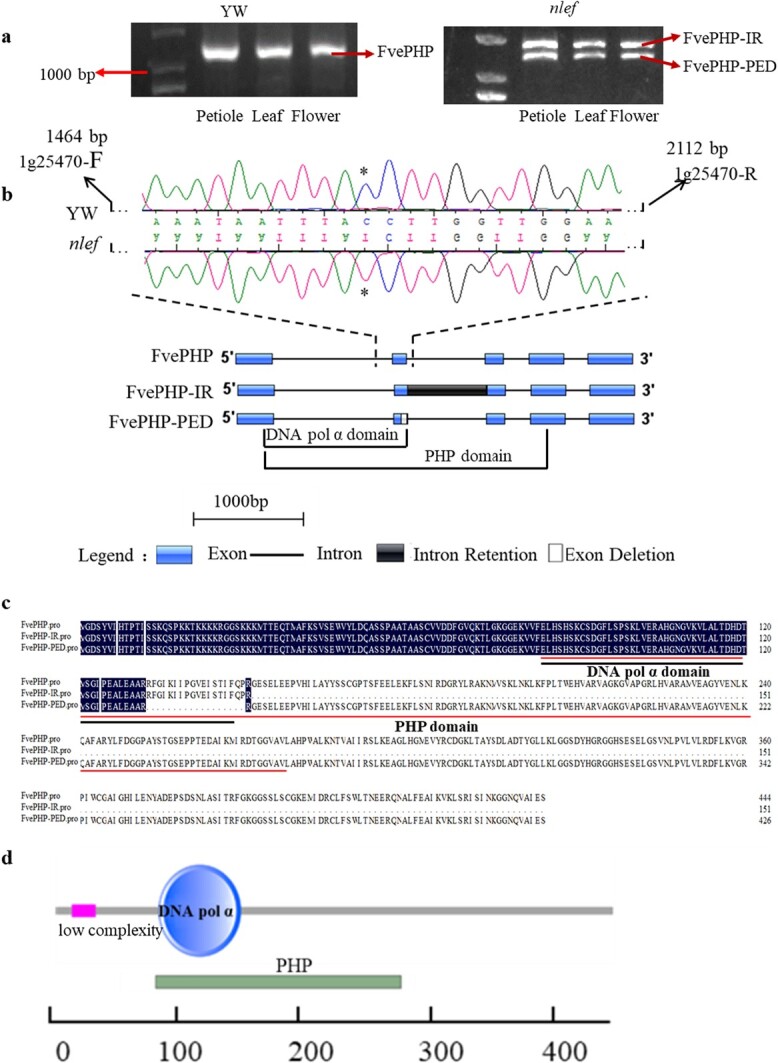
Bioinformatics analysis of the nucleotide and protein sequences of *FvePHP* and its mutant. **a** PCR amplification results for stems, leaves, and flowers in YW and *nlef* mutant. Electrophoresis bands in YW represent FvePHP, and in the mutant represent FvePHP-IR and FvePHP-PED. **b** Transcript structure and mutation and primer location. The asterisks represents the position of the mutation. The location of the primer is 1464 and 2112 bp for 1g25470-F and 1g25470-R, respectively. **c** Protein sequence alignment to FvePHP, FvePHP-IR, and FvePHP-PED. The asterisks represents the position of active sites involved in metal chelation. **d** Schematic diagram of the conserved domains of FvePHP and the DNA pol α domain predicted using SMART (http://smart.embl-heidelberg.de/).

To further investigate the mutation of the splice site, specific primers were designed with sizes between 1464 and 2112 bp for PCR amplification using DNA from the YW and mutant plants as templates. Sequencing of the PCR products indicated that the locus had a homozygous mutation (C in YW mutated to T in the mutant plants) (Fig. 5b). These results indicated that FvH4_1g25470 might be a candidate gene. Moreover, analysis of FvH4_1g25470 using the SMART website (http://smart.embl-heidelberg.de/) showed that FvH4_1g25470 encodes a hypothetical protein with one DNA polymerase α domain and one polymerase and histidinol phosphatase (PHP) domain (Fig. 5d). Herein, FvH4_1g25470 is named *FvePHP*.

### Cloning and bioinformatic analysis of *FvePHP*, *FvePHP-IR*, and *FvePHP-PED*

To identify the sequence differences, full-length CDSs from YW and the *nlef* mutant were amplified. The results showed one band in YW and two bands in the *nlef* mutant (Fig. 5a), and one band in the *nlef* mutant was larger than that in YW. Sequencing results showed two new transcripts in the *nlef* mutant due to mutations. One transcript contained one intron, suggesting an intron retention (IR) event in alternative splicing. In the other transcript, a part of an exon was found to be deleted (partial exon deletion, PED). Therefore, these transcripts were named *FvePHP-IR* and *FvePHP-PED*, respectively (Fig. 5b, Supplementary Data Fig. S2). According to genomic annotation information, *FvePHP* has five exons and four introns. Its full-length CDS has 1338 bp and encodes 445 amino acids and a termination codon. In mutant transcripts the full-length CDS and amino acids were altered. *FvePHP-IR* had a 456-bp CDS that encoded 151 amino acids and a termination codon in the second intron (Fig. 5c). *FvePHP-PED* had a 1281-bp CDS that encoded 427 amino acids and a termination codon, due to a 54-bp deletion in the second exon of *FvePHP* (Fig. 5c). These results suggest that the splice donor site mutation caused abnormal RNA splicing in *nlef*.

### Expression pattern and subcellular localization of *FvePHP*

**Figure 6:**
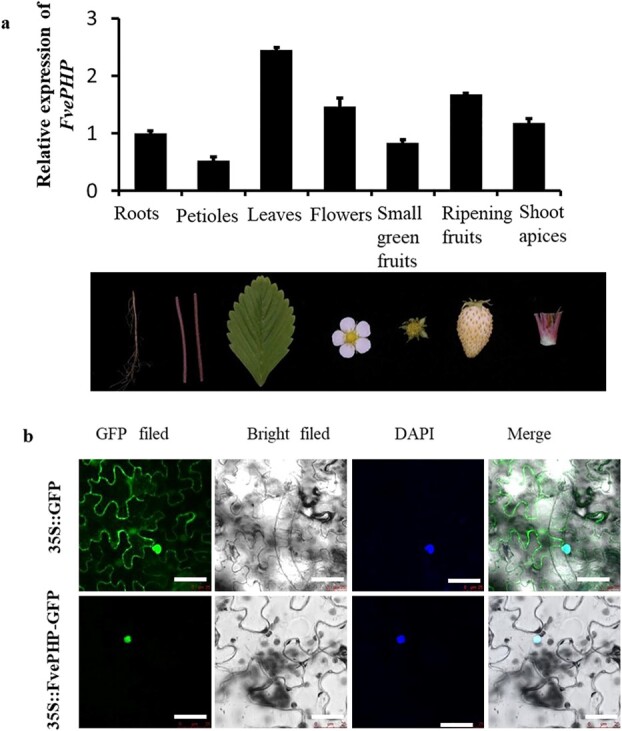
Expression pattern and subcellular localization of *FvePHP*. **a** Expression pattern analysis of *FvePHP* in different tissues and organs of YW. Error bars present the standard deviation. **b** Subcellular localization of the FvePHP protein. 35S::GFP was transformed into tobacco leaf epidermis and the GFP signal was detected in the nucleus and the cytoplasm (top). 35S::FvePHP-GFP was transformed into tobacco leaf epidermis (bottom), and the fusion protein signal was observed to be located in the nucleus. Scale bar = 25 μm.

To explore the temporal and spatial expression of *FvePHP* in YW plants, we conducted reverse transcription PCR (RT–qPCR) analysis in samples from the roots, petioles, leaves, flowers, small green fruits, ripening fruits, and shoot apices of YW. The results showed that the expression of *FvePHP* in YW leaves was higher than that in other tissues (Fig. 6a). The online website https://predictprotein.org/ [25] was used to predict the subcellular location of FvePHP, FvePHP-IR, and FvePHP-PED. Based on the prediction results, all three proteins, FvePHP, FvePHP-IR, and FvePHP-PED, were localized in the nucleus, with a prediction confidence score of 47, 72, and 60, respectively (Supplementary Data Fig. S2c). Furthermore, using the NLS_Mapper website (http://nls-mapper.iab.keio.ac.jp/cgi-bin/NLS_Mapper) [26–28], the signal peptides of nuclear localization signals located in the N termini of proteins were successfully predicted in three proteins (Supplementary Data Fig. S2b). To verify the subcellular location of the FvePHP protein with confidence score of 47, we transiently expressed the 35S::FvePHP-GFP fusion gene in tobacco leaf epidermal cells. Confocal microscopy was used to detect the protein signals. As shown in Fig. 6b, the fusion protein signal of FvePHP-GFP was colocalized with 6-diamidino-2-phenylindole in the nucleus of the epidermal cells of tobacco leaves, while the signal of 35S::GFP was distributed in the nucleus and the cytoplasm. All the above results indicated that *FvePHP* is expressed in all tissues and implies that its protein functions in the nucleus. Therefore, we speculate that FvePHP-IR and FvePHP-PED with higher confidence scores are also localized in the nucleus.

### Virus-induced gene silencing in ‘Yellow Wonder’ plants

**Figure 7:**
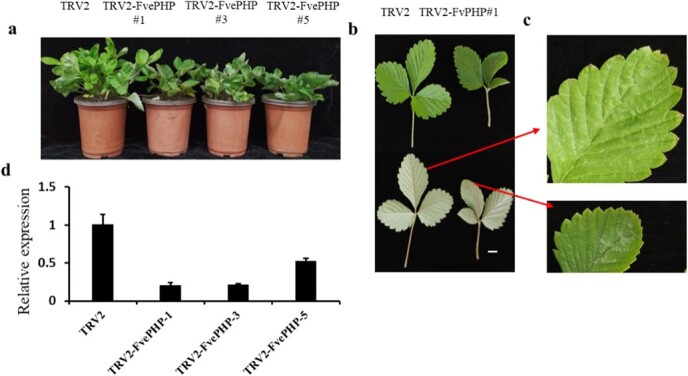
Virus-induced gene silencing of *FvePHP* in YW plants. **a** Phenotype of TRV2-FvePHP plants. Three TRV2*-*FvePHP plants showed a dwarf phenotype. **b** Leaf characteristics of TRV2 and TRV2*-*FvePHP #1 plants. **c** Leaflet margin characteristics of TRV2 and TRV2*-*FvePHP #1 plants. **d** Relative expression level analysis of *FvePHP* in silenced plants. The housekeeping gene *Fve26S* was used as control*.* All plants had been infected with *A. tumefaciens* 3 months previously. Scale bar = 1 cm.

To confirm the hypothesis that the low expression level of *FvePHP* might be a major cause for the developmental defects in the *nlef* mutant, virus-induced gene silencing (VIGS) assays were performed in the YW plants. Tobacco rattle virus (TRV) vector was used to carry out this experiment. *Agrobacterium tumefaciens* GV3101 carrying TRV2-FvePHP and TRV1 was injected into 1-month-old leaves of YW plants, and a mixture of TRV2 and TRV1 was used as a control. Three transformed plants were obtained with an obvious phenotype 3 months after infection by *A. tumefaciens* (Fig. 7a). As shown in Fig. 7a, the plant height of TRV2-FvePHP #1, #3, and #5 plants was lower than that of the control, and the number of serrations in the leaf margin was not significantly different (Fig. 7b and c). The leaves of the three transformed plants showed abnormal development, as shown in Fig. 7b. RT–qPCR results shown that expression levels of *FvePHP* were significantly reduced in silenced plants (Fig. 7d). TRV2-FvePHP lines did not display the same phenotype as mutant plants, which could be attributed to the low efficiency of VIGS in strawberries.

### Overexpression of *FvePHP* in *nlef* mutant recovered leaf phenotype and delayed flowering

**Figure 8:**
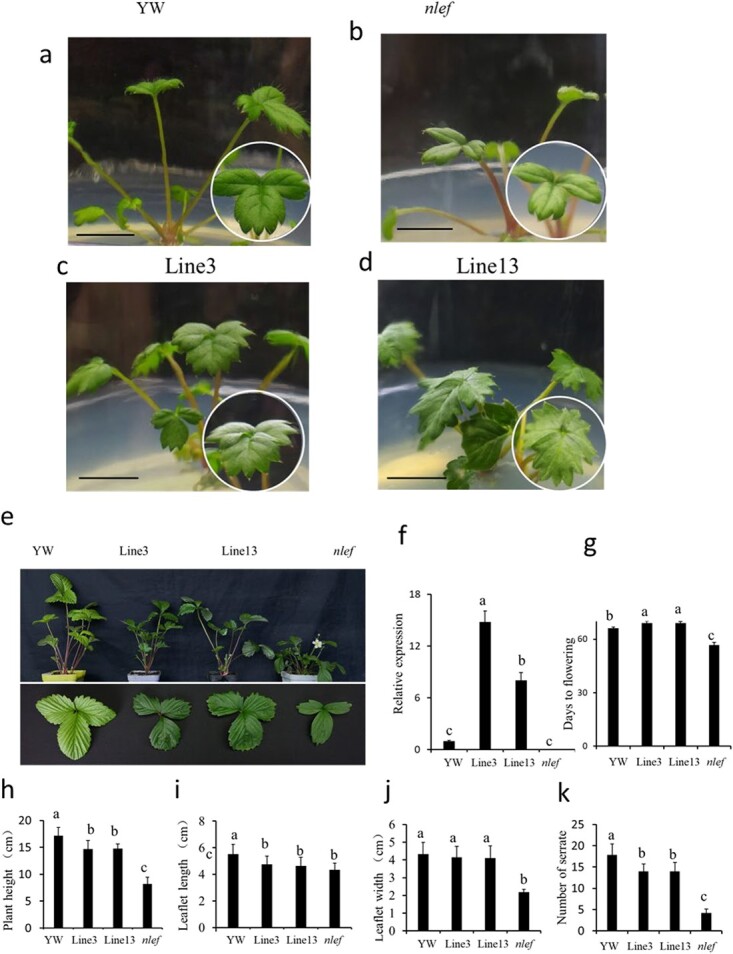
Phenotypes and gene expression levels of transgenic strawberry plants. **a**, **b** YW and mutant plants on media. **c**, **d** Two independent transgenic lines on media. **e** Phenotype of YW, *nlef* and transgenic plants grown in chamber conditions. **f** Relative expression level of *FvePHP*. Vertical bars represent the standard deviation (*n* = 3). **g** Flowering time of transgenic plants, mutants, and YW. **h**–**k** Morphological indicators of plant height, number of serrations, leaflet width, and leaflet length*.* Significance of differences was determined by the Duncan method (*P* = .05). Vertical bars represent the standard deviations of four plants. Scale bar = 0.5 cm (**a**–**d**).

To further confirm the phenotypes of *nlef* caused by a mutation of *FvePHP*, a complementation test was conducted. The full-length CDS of *FvePHP* was cloned into pRI101-AN (35S::FvePHP), and then transformed into the *nlef* mutant by *Agrobacterium*-mediated stable transformation with leaf pieces as explants. Two transgenic lines were obtained and identified by PCR (Supplementary Data Fig. S4). These transgenic lines on media have a similar phenotype, and they completely rescued the mutant defective in leaf serration (Fig. 8c and d).

In order to further observe the phenotype, two transgenic lines were transplanted in a chamber environment. To analyze the normal transcript level of FvePHP, specific primers from the flanks of the side of the second intron were designed (Supplementary Data Table S1). The relative expression level of *FvePHP* was analyzed using RT–qPCR in transgenic lines, YW and the *nlef* mutant. The transcript level of *FvePHP* was significantly increased in transgenic Lines 3 and 13 compared with YW and the *nlef* mutant (Fig. 8f). We further measured morphological traits, comprising plant height, leaflet length, leaflet width, and the serration number of the leaflet margin (Fig. 8h–k). Although plant height, leaflet width, and serration number of transgenic plants were significantly lower than those of YW, these indexes were significantly higher than those of the mutant. In addition, mutant plants started flowering at 55 days in the chamber, whereas the wild-type and *FvePHP*-overexpressing plants started flowering at 66 days (Fig. 8g, Supplementary Data Fig. S6). Further, the expression level of key flowering-time genes was analyzed (Supplementary Data Fig. S7). The expression of *FveAP1* was reduced in transgenic lines overexpressing *FvePHP* in the mutant compared with YW (Supplementary Data Fig. S7a and b). In addition, the expression of *FveTFL1* was lower in the transgenic plants compared with YW. The expression of *FveSOC1* was increased in transgenic Line 13 and similar to that in YW transgenic Line 3 (Supplementary Data Fig. S7c and d). These data showed no great differences in the expression of *FveAP1*, *FveTFL1*, and *FveSOC1* between transgenic plants and YW (Supplementary Data Fig. S7). The above results demonstrated that transgenic plants largely rescued the phenotypes of *nlef* plants.

### Expression analysis of flowering-related genes

**Figure 9:**
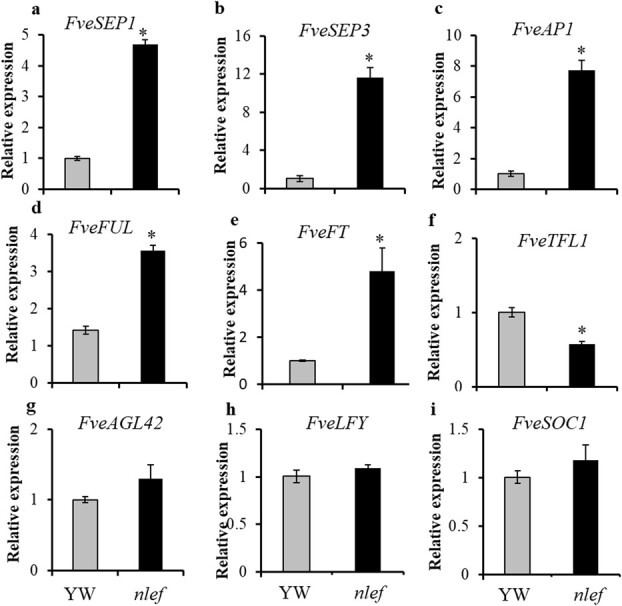
Expression analysis of flowering-related genes. RT–qPCR analysis of genes involved in flowering, including *FveSEP1*, *FveSEP3*, *FveAP1*, *FveTFL1*, *FveFUL*, *FveAGL42*, *FveLFY*, and *FveSOC1* in shoot apical meristem, and *FveFT* in leaf of YW and *nlef* mutant. *Fve26S* was used as the reference gene. Statistical significance was determined using Student’s *t*-test in different samples, ^*^P < .05. Error bars represent the standard deviation (*n* = 3).

To better explore the reason for early flowering in the *nlef* mutant, RT–qPCR was used to investigate several key flowering-related genes [29] in YW and the *nlef* mutant. Higher expression of positive regulators of flowering, *FveSEP1*, *FveSEP3*, *FveAP1*, *FveFUL*, and *FveFT*, was found in the mutant (Fig. 9a–e). The expression levels of *FveAGL42*, *FveLFY*, and *FveSOC1* were similar to that of YW (Fig. 9g–i). TERMINAL FLOWER1 (TFL1) is a flowering repressor in seasonal flowering accessions and FveTFL1 in YW is not functional [30, 31]. Surprisingly, we found that the mRNA expression level was decreased in the mutant compared with YW (Fig. 9f). These results suggest that several flowering-related genes are highly expressed, leading to early flowering phenotype in the *nlef* mutant.

## Discussion

In this study we identified a mutant, *nlef*, with narrow leaflets and an early-flowering phenotype in woodland strawberry (Fig. 1). We have provided evidence to support *FvePHP* as a candidate gene that encodes a putative α DNA polymerase. The phenotypes of *nlef* were similar to those of *A. thaliana* DNA polymerase α mutants *icu2* and *esd7* [32, 33], especially in the early flowering phenotype. In the 1.9-Mb region of Fvb1, only *FvePHP* showed a splicing event caused by a mutation of the 5′ splice site (Fig. 4e, Supplementary Data Fig. S1). The dCAPs marker of this SNP showed co-segregation with the mutant (Fig. 4c). Furthermore, the VIGS experiment indicated that low expression of *FvePHP* caused leaf development defects. The complementation assay revealed that overexpressing *FvePHP* in the mutant can restore the leaf phenotype and flowering. Overall, these results indicate that *FvePHP* is the candidate gene in the *nlef* mutant.

Cloning and sequencing of full-length cDNA indicated that the mutation at the splice site at the 5′ end of the second intron led to a mis-splicing event affecting the PHP domain of the polymerase. Intron retention would lead to more than loss of function of the PHP domain. In the spliceosome, the U1 snRNP is expected to recognize the 5′ splice site of the intron, and recruit the U2 snRNP to cut out the intron [32,33]. In FvePHP-IR, U1 could not recognize the mutated 5′ splice site, and the intron is retained, which results in the premature stop codon and loss of the 3′ end of the protein. Meanwhile, exon deletion also led to protein function loss of FvePHP. In the spliceosome, U1 snRNP could recognize new 5′ splice site, and recruit the U2 snRNP to cut the sequence between two splice sites [32,33]. In FvePHP-PED, U1 snRNP recognized new 5′ splice site because of the original splice site mutation. This resulted in a 54-bp deletion in the CDS. Therefore, the splice site mutation results in mis-splicing and generates new transcripts.

In *Escherichia coli*, DNA polymerase III contains a PHP domain to maintain its stability and activity, and its mutations led to decreased DNA polymerase III activity [34]. In *Thermus thermophilus* the PHP domain ensures the activity of family X DNA polymerases in base excision repair, and mutations in the PHP domain decrease the repair ability of these polymerases [35]. Moreover, the PHP domain of family X DNA polymerases exhibits 3′–5′ exonuclease activity. Therefore, the conserved PHP domain plays an important role in maintaining DNA polymerase activity and stability [34]. In this study the PHP domain of FvePHP-IR was disrupted, while FvePHP-PED was a relatively complete protein that retained the active sites (Fig. 5c). These putative activation sites are highly conserved and are involved in metal chelation (Supplementary Data Fig. S3) [36]. Active sites of polymerase can coordinate the catalytic metal to ensure the integrity of the polymerase activity [34]. In FvePHP-PED, although the functionally important active sites of catalytic metals were present, the PHP domain was seriously damaged and we speculated that FvePHP-PED might have weak activity (Supplementary Data Fig. S3). In FvePHP-IR, not only the active sites but also the PHP domain was seriously damaged. In brief, alternative splicing generated new proteins that had non-functional or low functional activity in *nlef*.

Eukaryotic polymerases involved in the cell cycle process affect plant growth and development [37,38]. In *A. thaliana*, mutation in eukaryotic polymerase α/ICU2 influences the development of the SAM and the cell cycle [39]. It is known that the leaves originate from the SAM and leaf growth and development are closely influenced by the cell cycle [40,41]. In this study, the leaf epidermal cells were observed by scanning electron microscopy, which showed that the *nlef* mutant had significantly reduced cell size.

Eukaryotic polymerase plays a key role in DNA replication [42], DNA repair, genomic stability, plant growth, and developmental processes, transcriptional gene silencing [43], and epigenetic control [44], and ensures the stability of histone modification [45]. Reactive oxygen species (ROS) can be produced when DNA replication is blocked [46]. ROS are considered to be signaling molecules in response to stress [47], not only affecting cell proliferation and size, but also promoting plant flowering through promoting expression of the flowering genes *FT1* and *FT2* in litchi leaves [48,49]. Besides, eukaryotic polymerase plays a key role in maintaining genomic stability; for example, *Arabidopsis* polymerase α ensures the stable maintenance of repressive histone modifications, and flowering genes *FT* and *AG* are upregulated because of loss of H3K27me3 in the DNA polymerase α mutant [45]. Eukaryotic polymerase δ can activate the epigenetic marks resulting in upregulation of the expression of *FT* and other flowering-related genes [39,50,51]. In *B. distachyon*, mutation of POLD3 causes the enrichment of the chromatin mark H3K4me3 in the flowering genes *VRN1*, *SEP3*, and *AG*, which leads to high expression of these flowering genes [22].

In this study the *nlef* mutant showed an early-flowering phenotype, and we found that the expression levels of *FveSEP1*, *FveSEP3*, *FveAP1*, *FveFUL*, and *FveFT* were significantly higher in the *nlef* mutant compared with YW. These results indicated that the *FvePHP* mutation activated the expression of flower-related genes, leading to an early-flowering phenotype. The relationship between FvePHP and histone modification of flowering genes in strawberry is unclear, so it needed to be explored further.

### Conclusions

In this study we identified a DNA polymerase gene (*FvePHP*) with a mutation at a splice site that produced two different transcripts, which led to the loss of function of *FvePHP.* On one hand, the *FvePHP* mutation caused multiple developmental defects, such as reduced plant size and leaf area. On the other hand, the mutation promoted the expression of several flowering-related genes leading to early flowering in strawberries.

## Materials and methods

### Plant materials and cultivation environments

Diploid woodland strawberry (*F. vesca*) ‘Yellow Wonder’ (YW) and ‘Ruegen’ (RG) were used in this study. The wild-type RG strawberry has red fruit and a red petiole and YW, with white fruit, is a natural *FvMYB10* gene mutant of RG. Mutant *nlef* was obtained from an ethyl methanesulfonate-mutated YW population. Selfing of the *nlef* generation was used to obtain the *M*_2_ generation. The *nlef* mutant plant was identified in the *M*_2_ generation and was crossed as the female parent to RG as the male parent. Self-pollination of *F*_1_-generation plants produced *F*_2_ populations. The phenotypes of the progeny from selfed *nlef* plants and the *F*_2_ population are shown in Supplementary Data Fig. S5. All plant materials were grown in a greenhouse at Shenyang Agricultural University in September. Seedling morphology was determined between 16 September and 15 October in a greenhouse at 26°C under 12-hours light/12-hours dark conditions.

Plant height, plant diameter, petiole length, leaflet length, and leaflet width were measured with a straightedge. The leaflet area was measured using ImageJ software. The third newly fully opened leaves were selected to measure the leaf shape traits.

Wild-type YW and mutant *nlef* seedlings were used for scanning electron microscopy and cultured in Murashige and Skoog (MS) medium at 23°C under long-day (16 hours light/8 hours dark) conditions*. Nicotiana benthamiana* was used for transient expression analysis and was grown at 23°C under long-day (16 hours light/8 hours dark) conditions for 1 month.

Early-flowering phenotypes were first investigated in a greenhouse with natural light and photographed. To further determine the flowering time, 70 mutants and YW plants were investigated in a chamber with mixed fluorescent blue and red light (95.96 μmol/m^2^/s), at 23–25°C under mid-day (12 hours light/12 hours dark) conditions.

### Scanning electron microscope analysis

Young leaves (3 mm × 5 mm) were fixed in 2.5% glutaraldehyde solution at 4°C for 48 hours according to a protocol by Lin *et al*. [52] The samples were then observed under a scanning electron microscope (Regulus 8100, Hitachi, Japan) at the Analytical and Testing Center, Shenyang Agricultural University, Shenyang. Cell area was determined with ImageJ software.

### DNA extraction and whole-genome sequencing

Genomic DNA from RG, *nlef* mutant, and 37 RG phenotype and 37 *nlef* phenotype plants from the *F*_2_ population was extracted using the modified cetyltrimethylammonium bromide (CTAB) method [53]. DNA concentration was determined using a NanoDrop 2000 spectrophotometer (Thermo Scientific) (concentration > 30 ng/μl, total amount of DNA ≥2 μg, OD_260/280_ = 1.7–2.2). Qualified libraries were sequenced at the Biomarker Technologies Corporation (Beijing, China) on a HiSeq X-Ten platform (Illumina, CA, USA).

### Read alignment, SNP/InDel variant detection, and annotation

Raw reads were qualified by removing low-quality reads (proportion of non-called bases >5%). Then, the clean reads were aligned to the *F. vesca* Genome v4.0.a1 [54] (https://www.rosaceae.org/species/fragaria/fragaria_vesca) using BWA software [54]. The detection and annotation of SNPs and InDels were performed using the Genome Analysis Toolkit [55] (GATK, https://software.broadinstitute.org/gatk/) and SnpEff [56], respectively.

### Identification and analysis of SNPs/InDels

A method using Euclidean distance (ED) [57] was employed to assess the association of these SNPs and InDels. The ED value was calculated as follows:

$$ \begin{align*} &\mathrm{ED}=\\ & \sqrt{{\left( Anlef\!-A\mathrm{RG}\right)}^2\!+{\left( Gnlef\!-G\mathrm{RG}\right)}^2\!+{\left( Cnlef\!-C\mathrm{RG}\right)}^2\!+{\left( Tnlef\!-T\mathrm{RG}\right)}^2} \end{align*}$$

where the four letters A, G, C, and T represent the frequency of the corresponding DNA nucleotides in the RG pool and *nlef* pool. The ED value of non-target sites should tend to 0. The higher the ED value, the more closely the variant is related to target sites.

### Primer design of InDel and dCAPs markers

Primer 5 software [58] was used to design the InDel marker primers. The primers for the dCAPs markers were designed using dCAPs Finder 2.0 (http://helix.wustl.edu/dcaps/dcaps.html). The primers used are shown in Supplementary Data Table S1.

### RNA extraction and gene expression analysis

Total RNA was extracted using the CTAB method [53]. RNA (1 μg) was reverse-transcribed in a 20-μl reaction using the PrimeScript™ RT Reagent Kit with gDNA Eraser (RR047a, TaKaRa, Otsu, Japan). The reaction was performed according to the manufacturer’s instructions. The cDNA obtained by reverse transcription was diluted five times and used in a real-time PCR experiment in an ABI 7500 system (Applied Biosystems, Foster City, CA, USA) performed with 10 μl of the reaction mix, which included 0.5 μl cDNA, 5 μl UltraSYBR Green Mixture reagent (CoWin Biotech, Beijing), 0.4 μl primer, and 4.1 μl ddH_2_O, and the relative expression levels were calculated using the 2^−ΔΔct^ method [59]. Samples were obtained from six YW and mutant plants. Each sample was quantified in triplicate. The RT–qPCR steps were as follows: 50°C for 2 minutes, 95°C for 10 minutes; 95°C for 15 seconds, 60°C for 1 minute; 40 cycles of 95°C for 15 seconds, 60°C for 15 seconds, and 95°C for 15 seconds. *Fve26S* was used as the control. Primers for flowering-related genes were obtained from a previously reported study by Lei *et al*. [29].

### Gene cloning and structure analysis

Full-length CDSs of YW and *nlef* were amplified using the primers listed in Supplementary Data Table S1. The PCR products were detected by 1.5% agarose gel electrophoresis and purified using a gel extraction kit (TIANGEN Biotech, Beijing). The PCR products were ligated into the pEASYT1 vector, and Sanger sequencing was performed. The peak values of the sequencing sites were observed using DNASTAR. Sequence alignment was performed using DNAMAN V6 (Lynnon BioSoft, Canada).

### Subcellular localization

The full-length CDS of *FvePHP* without the termination codon was amplified using specific primers with restriction sites (BamHI and KpnI) and then ligated to the pRI101-GFP vector by digestion. The pRI101-FvePHP-GFP and pRI101-GFP vectors were introduced into tobacco leaves via *Agrobacterium*-mediated transformation. The GFP fluorescence signal was detected and imaged using a laser confocal fluorescence microscope (TCS SP8-SE; Leica, Wetzlar, Germany). The DNA dye 4′,6-diamidino-2-phenylindole was used to label the cells to visualize the nucleus.

### Virus-induced gene silencing in wild-type plants

To verify the function of *FvePHP*, a 501-bp cDNA fragment of *FvePHP* was cloned into the BamHI and SacI sites of the pTRV2 vector. The method was based on a protocol by Lu *et al*. [60] with slight modifications as follows. The recombinant vector was named pTRV2-FvePHP. The plasmids pTRV1, pTRV2, and pTRV2-FvePHP were transformed into *A. tumefaciens* GV3101. GV3101-pTRV1, GV3101-pTRV2, and GV3101-pTRV2-FvePHP strains were grown in 5 ml yeast extract peptone (YEP) liquid medium (including 50 μg/ml kanamycin and 100 μg/ml rifampin) at 28°C for 24 hours at 180 rpm. Then, 1 ml of the bacterial liquid was transferred to 50 ml of YEP liquid medium followed by culture at 28°C for 6–8 hours at 180 rpm. The bacteria were collected (10 mM MES, 10 mM MgCl_2_, and 200 mM acetosyringone) by centrifugation and the OD_600_ value was adjusted to 1.2–1.5 using a spectrophotometer. GV3101-pTRV2 and GV3101-pTRV2-FvePHP were mixed with GV3101-pTRV1 at a 1:1 ratio for 1–2 hours at 28°C. The bacterial liquid was injected into the leaf through a 1-ml syringe without a needle. All plants injected with bacterial liquid were cultured in the dark for 24 hours and then placed in an incubator at 23°C (12 hours/12 hours dark) until they were transplanted to a greenhouse at Shenyang Agricultural University on 20 August, and were regularly observed multiple times until 25 October.

### Overexpression vector construction and plant transformation

The full-length CDS of *FvePHP* was amplified by RT–PCR from YW and cloned into pRI101-AN to generate the overexpression plasmid p35S::FvePHP. The primers are shown in Supplementary Data Table S1. The plasmid was identified by sequencing. The plasmid was introduced into *Agrobacterium* strain GV3101 via the freeze–thaw method and transformed into *nlef* strawberry. The transformation method was as described previously [61]. After 8 months, the transgenic plants were rooted in ½ MS medium with 0.1 mg/l indole 3-butyric acid (IBA) for 1 month. Then, the transgenic plants were transplanted and grown under greenhouse conditions. The transgenic strawberry plants were examined by PCR and RT–qPCR at DNA and RNA levels, respectively.

### cDNA library construction and RNA sequencing

Total RNA was extracted from YW and *nlef* leaves using TRIzol. RNA integrity was assessed using the RNA Nano 6000 Assay Kit of the Agilent Bioanalyzer 2100 system (Agilent Technologies). Sequencing libraries were generated using the NEB Next^®^ Ultra™ RNA Library Prep Kit for Illumina^®^ (NEB, USA). Libraries were sequenced by Wuhan Frasergen Bioinformatics Co. Ltd on an Illumina HiSeq X-Ten platform (Illumina, CA, USA) and 150-bp paired-end reads were generated.

### Reference sequence alignment

The raw sequencing data (raw reads) were filtered by removing the read adaptor and low-quality bases. Then clean data were obtained. Cleaned RNA-seq reads were mapped to the *F. vesca* whole genome [62] v4.0.a1 (https://www.rosaceae.org/species/fragaria_vesca/genome_v4.0.a1) using Bowtie 2 [63].

### Differential gene expression analysis

Fragments per kilobase per million bases (FPKM) was used to represent the level of gene expression [64]. Differentially expressed genes were analyzed using the R language package DEseq2, and differential genes were identified based on the FDR and fold change (logFc). Here, the screening standard was FDR <.05 and log_2_FC value >1 or <−1.

### Statistical analysis

Statistical analysis was performed using DPS 9.01 software. Statistical significance was tested using Student’s *t*-test, and the significance level was set at ^*^P < .05 and ^**^P < .01.

## Acknowledgements

We express our gratitude to the anonymous reviewers for helpful comments to improve the manuscript. This work was financially supported by Liao Ning Revitalization Talents Program (No. XLYC1902069).

## Author contributions

J.Z. and Z.Z. conceived and designed this research. B.W., W.L., Y.L., D.Z., K.X., and X.L. conducted the experiments. B.W., J.Z., and Z.Z. wrote and modified the manuscript. All authors involved in this study read and approved the manuscript.

## Data availability

The BSA sequence data have been deposited in the Genome Warehouse in the National Genomics Data Center, Beijing Institute of Genomics (BIG), Chinese Academy of Sciences, under accession number CRA005241, and are publicly accessible at https://bigd.big.ac.cn/gwh. RNA-seq data were submitted to NCBI and the accession number is PRJNA597925.

## Conflict of interest

The authors have no conflict of interest to declare.

## Supplementary data

Supplementary data is available at *Horticulture Research* online.

## Supplementary Material

Web_Material_uhac249

## References

[1] Yuan HZ , Yu H, Huang T et al. The complexity of the *Fragaria × ananassa* (octoploid) transcriptome by single-molecule long-read sequencing. *Hortic Res*. 2019;6:46. 30962939 10.1038/s41438-019-0126-6 PMC6441658

[2] Efroni I , Eshed Y, Lifschitz E. Morphogenesis of simple and compound leaves: a critical review. *Plant Cell*. 2010;22:1019–32. 20435903 10.1105/tpc.109.073601 PMC2879760

[3] Bernier G . The control of floral evocation and morphogenesis. *Annu Rev Plant Biol*. 1988;39:175–219.

[4] Chitwood DH , Sinha NR. Evolutionary and environmental forces sculpting leaf development. *Curr Biol*. 2016;26:R297–306. 27046820 10.1016/j.cub.2016.02.033

[5] Mohammed B , Bilooei SF, Dóczi R et al. Converging light, energy and hormonal signaling control meristem activity, leaf initiation, and growth. *Plant Physiol*. 2018;176:1365–81. 29284741 10.1104/pp.17.01730 PMC5813583

[6] Liu HP , Yu HY, Tang GL et al. Small but powerful: function of microRNAs in plant development. *Plant Cell Rep*. 2018;37:515–28. 29318384 10.1007/s00299-017-2246-5

[7] Bresso EG , Chorostecki U, Rodriguez RE et al. Spatial control of gene expression by mir319-regulated TCP transcription factors in leaf development. *Plant Physiol*. 2018;176:1694–708. 29133375 10.1104/pp.17.00823 PMC5813565

[8] Rodriguez RE , Debernardi JM, Palatnik JF. Morphogenesis of simple leaves: regulation of leaf size and shape. *Wiley Interdiscip Rev Dev Biol*. 2014;3:41–57. 24902833 10.1002/wdev.115

[9] Yasui Y , Ohmori Y, Takebayashi Y et al. WUSCHEL-RELATED HOMEOBOX4 acts as a key regulator in early leaf development in rice. *PLoS Genet*. 2018;14:e1007365. 29684018 10.1371/journal.pgen.1007365 PMC5933814

[10] Scanlon MJ . Leaves of grass: focusing phenomics on maize leaf growth. *Genome Biol*. 2015;16:1–3. 26381482 10.1186/s13059-015-0773-3 PMC4574536

[11] Jiang FK , Guo M, Yang F et al. Mutations in an AP2 transcription factor-like gene affect internode length and leaf shape in maize. *PLoS One*. 2012;7:e37040. 22649507 10.1371/journal.pone.0037040 PMC3359370

[12] Wu LJ , Tian ZD, Zhang JH. Functional dissection of auxin response factors in regulating tomato leaf shape development. *Front Plant Sci*. 2018;9:957. 30022995 10.3389/fpls.2018.00957 PMC6040142

[13] Imriani PS , Yano R, Okabe Y et al. SlLAX1 is required for normal leaf development mediated by balanced adaxial and abaxial pavement cell growth in tomato. *Plant Cell Physiol*. 2018;59:1170–86. 29528453 10.1093/pcp/pcy052

[14] Liu MY , Lei L, Miao F et al. The STENOFOLIA gene from *Medicago* alters leaf width, flowering time and chlorophyll content in transgenic wheat. *Plant Biotechnol J*. 2018;16:186–96. 28509374 10.1111/pbi.12759 PMC5785358

[15] Rowland SD , Zumstein K, Nakayama H et al. Leaf shape is a predictor of fruit quality and cultivar performance in tomato. *New Phytol*. 2020;226:851–65. 31880321 10.1111/nph.16403 PMC7187315

[16] Hytönen T , Kurokura T. Control of flowering and runnering in strawberry. *Hortic J*. 2020;89:96–107.

[17] Farrona S , Coupland G, Turck F. The impact of chromatin regulation on the floral transition. *Semin Cell Dev Biol*. 2008;19:560–73. 18708152 10.1016/j.semcdb.2008.07.015

[18] Tan LM , Liu R, Gu BW et al. Dual recognition of H3K4me3 and DNA by the ISWI component ARID5 regulates the floral transition in *Arabidopsis*. *Plant Cell*. 2020;32:2178–95. 32358072 10.1105/tpc.19.00944 PMC7346560

[19] Szakonyi D , Duque P. Alternative splicing as a regulator of early plant development. *Front Plant Sci*. 2018;9:1174. 30158945 10.3389/fpls.2018.01174 PMC6104592

[20] Staiger D , Brown JW. Alternative splicing at the intersection of biological timing, development, and stress responses. *Plant Cell*. 2013;25:3640–56. 24179132 10.1105/tpc.113.113803 PMC3877812

[21] Zuo Y , Feng F, Qi WW et al. Dek42 encodes an RNA-binding protein that affects alternative pre-mRNA splicing and maize kernel development. *J Integr Plant Biol*. 2019;61:728–48. 30839161 10.1111/jipb.12798

[22] Woods DP , Dong Y, Bouché F et al. Mutations in the predicted DNA polymerase subunit POLD3 result in more rapid flowering of *Brachypodium distachyon*. *New Phytol*. 2020;227:1725–35. 32173866 10.1111/nph.16546

[23] Santiago MJ , Alejandre-Duran E, Ruiz-Rubio M. Alternative splicing of two translesion synthesis DNA polymerases from *Arabidopsis thaliana*. *Plant Sci*. 2009;176:591–6. 26493150 10.1016/j.plantsci.2009.01.018

[24] Wang X , Gao D, Sun J et al. An exon skipping in a SEPALLATA-like gene is associated with perturbed floral and fruits development in cucumber. *J Integr Plant Biol*. 2016;58:766–71. 26936301 10.1111/jipb.12472

[25] Bernhofer M , Dallago C, Karl T et al. PredictProtein – predicting protein structure and function for 29 years. *Nucleic Acids Res*. 2021;49:W535–40. 33999203 10.1093/nar/gkab354 PMC8265159

[26] Kosugi S , Hasebe M, Tomita M et al. Systematic identification of yeast cell cycle-dependent nucleocytoplasmic shuttling proteins by prediction of composite motifs. *Proc Natl Acad Sci USA*. 2009;106:10171–6. 19520826 10.1073/pnas.0900604106 PMC2695404

[27] Kosugi S , Hasebe M, Matsumura N et al. Six classes of nuclear localization signals specific to different binding grooves of importin α. *J Biol Chem*. 2009;284:478–85. 19001369 10.1074/jbc.M807017200

[28] Kosugi S , Hasebe M, Entani T et al. Design of peptide inhibitors for the importin α/β nuclear import pathway by activity-based profiling. *Chem Biol*. 2008;15:940–9. 18804031 10.1016/j.chembiol.2008.07.019

[29] Lei YY , Sun Y, Wang B et al. Woodland strawberry *WRKY71* acts as a promoter of flowering via a transcriptional regulatory cascade. *Hortic Res*. 2020;7:137. 32922809 10.1038/s41438-020-00355-4 PMC7458929

[30] Koskela EA , Mouhu K, Albani MC et al. Mutation in TERMINAL FLOWER1 reverses the photoperiodic requirement for flowering in the wild strawberry *Fragaria vesca*. *Plant Physiol*. 2012;159:1043–54. 22566495 10.1104/pp.112.196659 PMC3387692

[31] Joldersma D , Sadowski N, Timp W et al. Assembly and annotation of *Fragaria vesca* ‘Yellow Wonder’ genome, a model diploid strawberry for molecular genetic research. *Fruit Res*. 2022;2:13.

[32] Wahl MC , Will CL, Lührmann R. The spliceosome: design principles of a dynamic RNP machine. *Cell*. 2009;136:701–18. 19239890 10.1016/j.cell.2009.02.009

[33] Conti LD , Baralle M, Buratti E. Exon and intron definition in pre-mRNA splicing. *Wiley Interdiscip Rev RNA*. 2013;4:49–60. 23044818 10.1002/wrna.1140

[34] Barros T , Guenther J, Kelch B et al. A structural role for the PHP domain in *E. coli* DNA polymerase III. *BMC Struct Biol*. 2013;13:8. 23672456 10.1186/1472-6807-13-8 PMC3666897

[35] Nakane S , Nakagawa N, Kuramitsu S et al. The role of the PHP domain associated with DNA polymerase X from *Thermus thermophilus* HB8 in base excision repair. *DNA Repair*. 2012;11:906–14. 23068311 10.1016/j.dnarep.2012.09.001

[36] Aravind L , Koonin EV. Phosphoesterase domains associated with DNA polymerases of diverse origins. *Nucleic Acids Res*. 1998;26:3746–52. 9685491 10.1093/nar/26.16.3746 PMC147763

[37] Roy S , Choudhury SR, Singh SK et al. AtPolλ, a homolog of mammalian DNA polymerase λ in *Arabidopsis thaliana*, is involved in the repair of UV-B induced DNA damage through the dark repair pathway. *Plant Cell Physiol*. 2011;52:448–67. 21227935 10.1093/pcp/pcr002

[38] Dewar JM , Walter JC. Mechanisms of DNA replication termination. *Nat Rev Mol Cell Biol*. 2017;18:507–16. 28537574 10.1038/nrm.2017.42 PMC6386472

[39] Barrero JM , González-Bayón R, del Pozo JC et al. INCURVATA2 encodes the catalytic subunit of DNA polymerase α and interacts with genes involved in chromatin-mediated cellular memory in *Arabidopsis thaliana*. *Plant Cell*. 2007;19:2822–38. 17873092 10.1105/tpc.107.054130 PMC2048701

[40] Dengler NG , Tsukaya H. Leaf morphogenesis in dicotyledons: current issues. *Int J Plant Sci*. 2001;162:459–64.

[41] Horiguchi G , Ferjani A, Fujikura U et al. Coordination of cell proliferation and cell expansion in the control of leaf size in *Arabidopsis thaliana*. *J Plant Res*. 2006;119:37–42. 16284709 10.1007/s10265-005-0232-4

[42] Hübscher U , Nasheuer HP, Syväoja JE. Eukaryotic DNA polymerases, a growing family. *Trends Biochem Sci*. 2000;25:143–7. 10694886 10.1016/s0968-0004(99)01523-6

[43] Liu J , Ren X, Yin H et al. Mutation in the catalytic subunit of DNA polymerase alpha influences transcriptional gene silencing and homologous recombination in *Arabidopsis*. *Plant J*. 2010;61:36–45. 19769574 10.1111/j.1365-313X.2009.04026.x

[44] Nakayama JI , Allshire RC, Klar AJ et al. A role for DNA polymerase α in epigenetic control of transcriptional silencing in fission yeast. *EMBO J*. 2001;20:2857–66. 11387218 10.1093/emboj/20.11.2857 PMC125490

[45] Hyun Y , Yun H, Park K et al. The catalytic subunit of *Arabidopsis* DNA polymerase α ensures stable maintenance of histone modification. *Development*. 2013;140:156–66. 23154417 10.1242/dev.084624

[46] Matic I . The major contribution of the DNA damage-triggered reactive oxygen species production to cell death: implications for antimicrobial and cancer therapy. *Curr Genet*. 2018;64:567–9. 29181628 10.1007/s00294-017-0787-3

[47] Hatano-Iwasaki A , Ogawa K. Redox metabolism in response to environmental stimuli for flowering. *Funct Plant Sci Biotechnol*. 2007;1:246–53.

[48] Schippers JH , Foyer CH, van Dongen JT. Redox regulation in shoot growth, SAM maintenance and flowering. *Curr Opin Plant Biol*. 2016;29:121–8. 26799134 10.1016/j.pbi.2015.11.009

[49] Lu X , Yu S, Lü P et al. Genome-wide transcriptomic analysis reveals a regulatory network of oxidative stress-induced flowering signals produced in litchi leaves. *Genes*. 2020;11:324. 32197528 10.3390/genes11030324 PMC7140818

[50] Olmo ID , López-González L, Martín-Trillo MM et al. *EARLY IN SHORT DAYS 7(ESD7)* encodes the catalytic subunit of DNA polymerase epsilon and is required for flowering repression through a mechanism involving epigenetic gene silencing. *Plant J*. 2010;61:623–36. 19947980 10.1111/j.1365-313X.2009.04093.x

[51] Iglesias FM , Bruera NA, Dergan-Dylon S et al. The *Arabidopsis* DNA polymerase δ has a role in the deposition of transcriptionally active epigenetic marks, development and flowering. *PLoS Genet*. 2015;11:e1004975. 25693187 10.1371/journal.pgen.1004975 PMC4334202

[52] Lin S , Dong H, Zhang F et al. BcMF8, a putative arabinogalactan protein-encoding gene, contributes to pollen wall development, aperture formation and pollen tube growth in *Brassica campestris*. *Ann Bot*. 2014;113:777–88. 24489019 10.1093/aob/mct315 PMC3962243

[53] Chang L , Zhang Z, Yang H et al. Detection of strawberry RNA and DNA viruses by RT-PCR using total nucleic acid as a template. *J Phytopathol*. 2007;155:431–6.

[54] Li H , Durbin R. Fast and accurate short read alignment with Burrows-Wheeler transform. *Bioinformatics*. 2009;25:1754–60. 19451168 10.1093/bioinformatics/btp324 PMC2705234

[55] McKenna A , Hanna M, Banks E et al. The Genome Analysis Toolkit: a MapReduce framework for analyzing next-generation DNA sequencing data. *Genome Res*. 2010;20:1297–303. 20644199 10.1101/gr.107524.110 PMC2928508

[56] Cingolani P , Platts A, Wang LL et al. A program for annotating and predicting the effects of single nucleotide polymorphisms, SnpEff: SNPs in the genome of *Drosophila melanogaster* strain w^1118^; iso-2; iso-3. *Fly*. 2012;6:80–92. 22728672 10.4161/fly.19695 PMC3679285

[57] Hill JT , Demarest BL, Bisgrove BW et al. MMAPPR: mutation mapping analysis pipeline for pooled RNA-seq. *Genome Res*. 2013;23:687–97. 23299975 10.1101/gr.146936.112 PMC3613585

[58] Abd-Elsalam KA . Bioinformatic tools and guideline for PCR primer design. *Afr J Biotechnol*. 2003;2:91–5.

[59] Bustin SA , Benes V, Garson JA et al. The MIQE guidelines: minimum information for publication of quantitative real-time PCR experiments. *Clin Chem*. 2009;55:611–22. 19246619 10.1373/clinchem.2008.112797

[60] Lu R , Martin-Hernandez AM, Peart JR et al. Virus-induced gene silencing in plants. *Methods*. 2003;30:296–303. 12828943 10.1016/s1046-2023(03)00037-9

[61] Li WJ , Zhang J, Sun H et al. FveRGA1, encoding a DELLA protein, negatively regulates runner production in *Fragaria vesca*. *Planta*. 2018;247:941–51. 29288326 10.1007/s00425-017-2839-9

[62] Edger PP , VanBuren R, Colle M et al. Single-molecule sequencing and optical mapping yields an improved genome of woodland strawberry (*Fragaria vesca*) with chromosome-scale contiguity. *Gigascience*. 2018;7:1–7. 10.1093/gigascience/gix124 PMC5801600 29253147

[63] Langmead B . Aligning short sequencing reads with Bowtie. *Curr Protoc Bioinformatics*. 2010;11.7.1–11.7.14. 10.1002/0471250953.bi1107s32 PMC3010897 21154709

[64] Trapnell C , Williams BA, Pertea G et al. Transcript assembly and quantification by RNA-Seq reveals unannotated transcripts and isoform switching during cell differentiation. *Nat Biotechnol*. 2010;28:511–5. 20436464 10.1038/nbt.1621 PMC3146043

