# Supplementary material for: A splice site mutation in the FvePHP gene is associated with leaf development and flowering time in woodland strawberry
Source: Hortic Res. 2022 Nov 10;10(1):uhac249. doi: 10.1093/hr/uhac249 (PMC9832950; doi:10.1093/hr/uhac249)
Supplement: Web_Material_uhac249 [file web_material_uhac249.zip › Supplementary Fig.S1-S7.doc]

**Fig. S1 Venn map of splicing event genes in the 1.94 Mb candidate regions.**

A is candidate gene from the BSA-seq. B and C are splicing event genes from RNA-seq of Yellow Wonder and *nlef* plants, respectively (the candidate region is 1.94Mb).

**Fig. S2 Sequence alignments of nucleic acids and amino acids of different transcripts FvePHP-IR, FvePHP and FvePHP-PED, respectively.** **a** The sequence alignment of intron retention of FvePHP-IR and deletion partial exon of FvePHP-PED. **b** The amino acid sequence alignment and nuclear localization signals (KTKKKRGGS) of the FvePHP-IR, FvePHP, and FvePHP-PED proteins. **c** Subcellular localization of the FvePHP, FvePHP-IR, and FvePHP-PED were predicted by the online website (<https://predictprotein.org/>). All three proteins FvePHP, FvePHP-IR, and FvePHP-PED were localized in the nucleus and the prediction confidence score was 47, 72, 60, respectively.  Confidence score (ranging from 0 = unreliable to 100 = reliable).

**Fig. S3 Proteins sequences alignment of FvePHP homologs from other species****.** Alignment of the PHP domains. AT2G13480, XP_018842988.1 (*Juglans regia*), XP_034229104.1 (*Prunus dulcis*), XP_008237177.1 (*Prunus mume*), PQM32927.1 (*Prunus yedoensis*) were obtained from the NCBI. The arrow indicated the active sites which were involved in metal chelation. Completely conserved amino acids in these species were represented black, and incompletely conserved amino acids were represented by pink.

**Fig. S4 DNA level identified of the transgenic lines** **overexpressing *FvePHP* in the mutant.**

**a** Schematic diagram of the over-expression vector. **b** Transgenic strawberry were identified by RT-PCR. The plasmid and p35S::FvePHP were used as positive control. Mutant strawberry *nlef* was used as negative control.

**Fig. S5 The phenotypes of selfed progeny of *nlef* mutant and F_2_ individuals from the cross between *nlef* and Ruegen. a** Phenotypic characteristics of mutant and its selfed progeny. **b** Phenotypic characteristics of F_1_, and offspring plants from F_1_ self-cross. The detail of leaves between wild type and mutant was shown the bottom of the Figure.

**Fig. S6 The phenotype of regeneration between YW and *nlef* (a) and the transgenic lines overexpressing FvePHP in the mutant, *nlef* plants for morphological characteristic (b)**. **a** Investigation of regeneration ability of YW and *nlef* plants. Leaf segment was observed after 75 days of incubation in regeneration medium (MS salts, 3% sucrose, 0.7% agar, 4.0 mg L^-1^ TDZ, and 0.1 mg L^-1^ IBA, pH = 5.4). **b** Morphological characteristics of transgenic plants and Yellow Wonder, mutant plants at 66 days after planting.

**Fig. S7 Gene expression levels of flowering time gene of *FveAP1*, *FveLFY* and *FveTFL1 FveSOC1* in control Yellow Wonder plants and transgenic lines overexpressing *FvePHP* in the mutant.** The shoot apical meristem from six plants were mixed from Yellow Wonder, transgenic Line3, transgenic Line13, for RNA extraction, respectively. Gene expression was examined in triplicate.
